# Supplementary figures and images for: Impact of physical activity on postural stability and coordination in children with posterior fossa tumor: randomized control phase III trial
Source: J Cancer Res Clin Oncol. 2022 Dec 16;149(9):5637–44. doi: 10.1007/s00432-022-04490-4 (PMC10356666; doi:10.1007/s00432-022-04490-4)

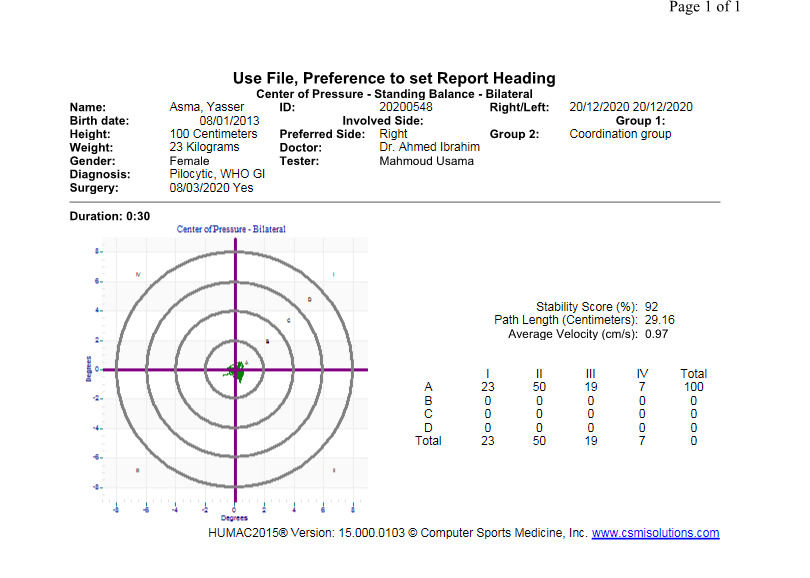

Supplement: Supplementary file 1 — Supplementary file1 (JPG 104 KB) [file 432_2022_4490_MOESM1_ESM.jpeg]

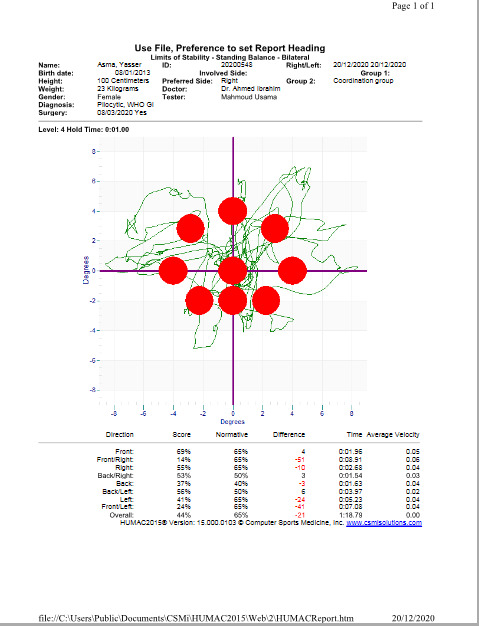

Supplement: Supplementary file 2 — Supplementary file2 (JPG 83 KB) [file 432_2022_4490_MOESM2_ESM.jpeg]

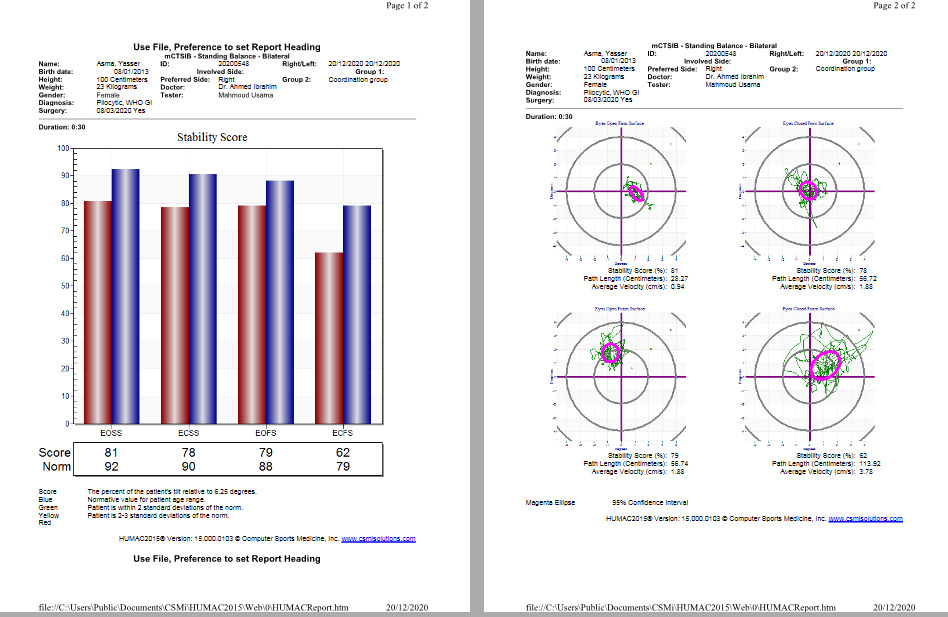

Supplement: Supplementary file 3 — Supplementary file3 (JPG 171 KB) [file 432_2022_4490_MOESM3_ESM.jpeg]

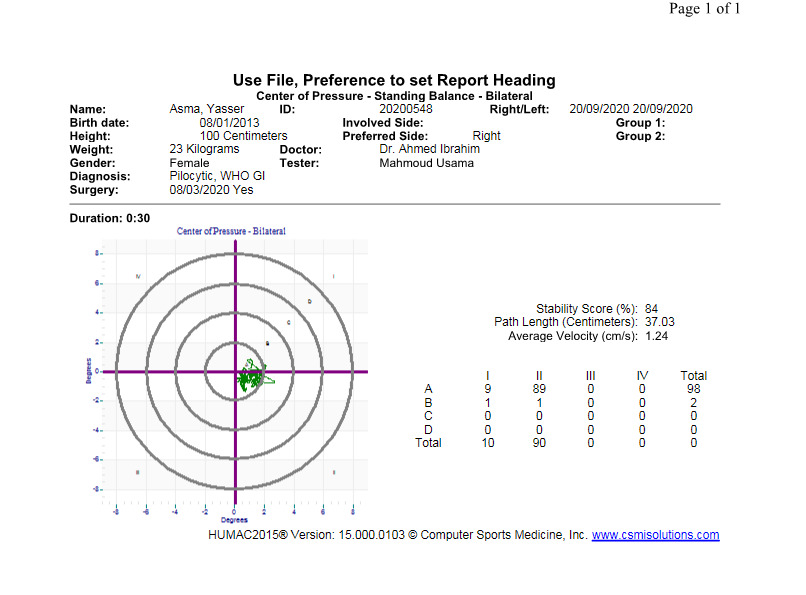

Supplement: Supplementary file 4 — Supplementary file4 (JPG 103 KB) [file 432_2022_4490_MOESM4_ESM.jpeg]

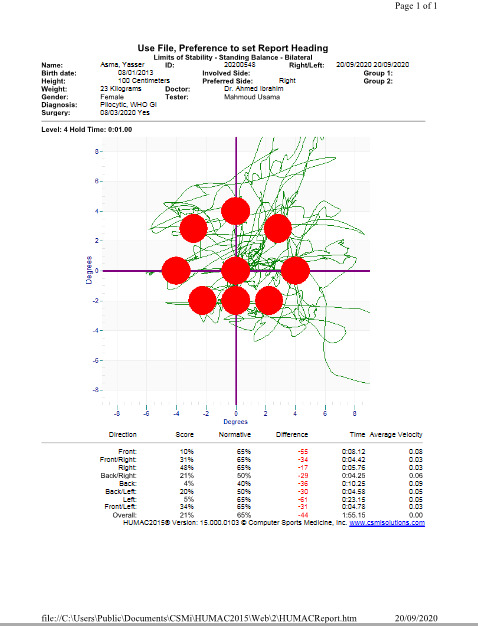

Supplement: Supplementary file 5 — Supplementary file5 (JPG 89 KB) [file 432_2022_4490_MOESM5_ESM.jpeg]

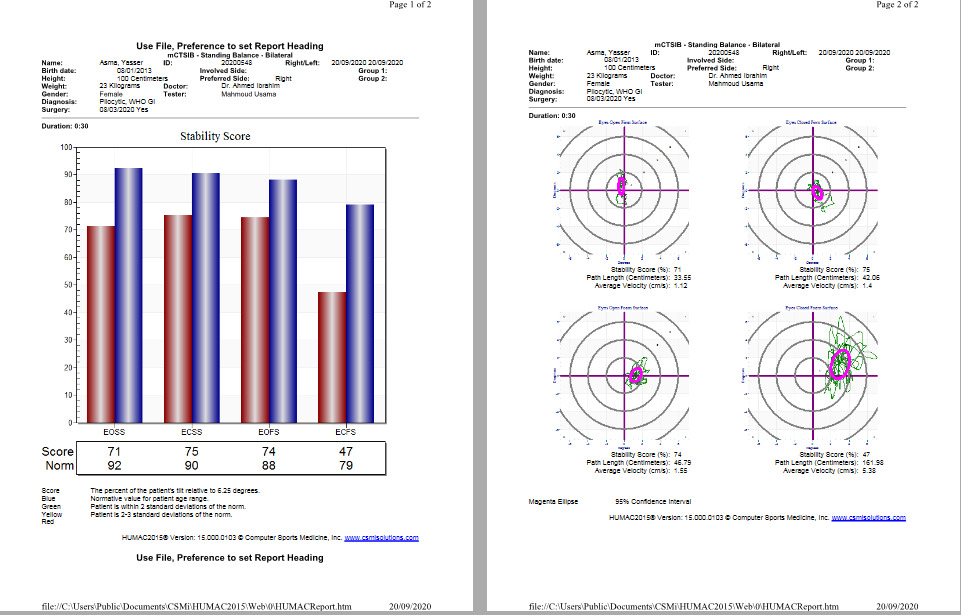

Supplement: Supplementary file 6 — Supplementary file6 (JPG 165 KB) [file 432_2022_4490_MOESM6_ESM.jpeg]
